# Crimean-Congo hemorrhagic fever virus in livestock ticks and animal handler seroprevalence at an abattoir in Ghana

**DOI:** 10.1186/s12879-016-1660-6

**Published:** 2016-07-08

**Authors:** R. Akuffo, J. A. M. Brandful, A. Zayed, A. Adjei, N. Watany, N. T. Fahmy, R. Hughes, B. Doman, S. V. Voegborlo, D. Aziati, D. Pratt, J. A. Awuni, N. Adams, E. Dueger

**Affiliations:** U.S Naval Medical Research Unit No. 3, Cairo, Egypt; Centers for Disease Control and Prevention, Atlanta, GA USA; Noguchi Memorial Institute for Medical Research, University of Ghana, Accra, Ghana; University of Ghana, Accra, Ghana; Veterinary Services of Ghana, Accra, Ghana; Present Address: NAMRU-3, PSC 452, P.O Box 5000, FPO, AE 09835-9998, 3A Imtidad Ramses Street. Adjacent to Abbassia Fever Hospital, Abbassia, Cairo Egypt

**Keywords:** Crimean-congo hemorrhagic fever virus, Crimean-congo hemorrhagic fever, Seroprevalence, Ticks, Ambylomma, Hyalomma, Boophilus, Livestock, Abattoir, Ghana

## Abstract

**Background:**

Crimean-Congo Haemorrhagic Fever Virus (CCHFV) is a zoonotic virus transmitted by Ixodid ticks and causes Crimean-Congo hemorrhagic fever (CCHF) disease in humans with up to 50 % mortality rate.

**Methods:**

Freshly slaughtered livestock at the Kumasi abattoir in the Ashanti Region of Ghana were examined for the presence of ticks once a month over a 6-month period from May to November 2011. The ticks were grouped into pools by species, sex, and animal source. CCHFV was detected in the ticks using reverse transcription PCR. Blood samples were collected from enrolled abattoir workers at initiation, and from those who reported fever in a preceding 30-day period during monthly visits 2–5 months after initiation. Six months after initiation, all participants who provided baseline samples were invited to provide blood samples. Serology was performed using enzyme linked immunosorbent assay (ELISA). Demographic and epidemiological data was also obtained from enrolled participants using a structured questionnaire.

**Results:**

Of 428 freshly slaughtered animals comprising 130 sheep, 149 cattle, and 149 goats examined, 144 ticks belonging to the genera *Ambylomma*, *Hyalomma* and *Boophilus* were identified from 57 (13.3 %): 52 (34.9 %), 4 (3.1 %) and 1 (0.7 %) cattle, sheep and goat respectively. Of 97 tick pools tested, 5 pools comprising 1 pool of *Hyalomma excavatum* and 4 pools of *Ambylomma variegatum,* collected from cattle, were positive for CCHFV. Of 188 human serum samples collected from 108 abattoir workers, 7 (3.7 %) samples from 6 persons were anti-CCHF IgG positive with one of them also being CCHF IgM positive. The seroprevalence of CCHFV identified in this study was 5.7 %.

**Conclusions:**

This study detected human exposure to CCHF virus in slaughterhouse workers and also identified the CCHF virus in proven vectors (ticks) of Crimean Congo hemorrhagic fever in Ghana. The CCHFV was detected only in ticks collected from cattle, one of the livestock known to play a role in the amplification of the CCHF virus.

## Background

Crimean-Congo Haemorrhagic Fever Virus (CCHFV) is a member of the *Nairovirus* genus of the family *Bunyaviridae* that can be transmitted to humans. This arbovirus is transmitted by Ixodid ticks and causes a highly pathogenic disease called Crimean Congo hemorrhagic fever (CCHF) with up to 50 % mortality rate in humans [1, 2]. At least 28 species of the Ixodid ticks distributed among 7 genera, (*Hyalomma*, *Rhipicephalus*, *Boophilus*, *Dermacentor*, *Ambylomma*, *Haemaphysalis*, and *Ixodes)* have been found to be naturally infected with CCHF virus worldwide [3, 4].

Crimean Congo hemorrhagic fever (CCHF) is endemic to Africa, the Balkans, the Middle East, and parts of Asia. Although the hard ticks (Ixodids) serve as reservoirs and vectors for CCHFV, a variety of animals, such as cattle, sheep, goat and camels, are considered amplifying hosts for the virus. The CCHF virus has been isolated from ticks, domestic and wild vertebrates, and humans in some sub-Saharan West African countries such as Senegal, Mauritania, and Burkina Faso [5–8].

The CCHFV can cause a severe disease in humans by exposure to bites from infected ticks or by crushing infected ticks with an open wound, contact with blood or tissues from infected patients and animals, and drinking unpasteurized milk from infected animals. Other documented modes of transmission include aerosol transmission as well as horizontal transmission from a mother to her child in Russia. However, the virus causes limited or no disease in their zoonotic hosts [9, 10].

Several factors influence CCHF morbidity and mortality. The incidence of the CCHF disease as well as the numbers of ticks in the environment is influenced by climatic factors, geographic conditions and the presence and habitat preferences of host animals. As a result, seasonality of the disease has been observed in some countries such as Iran and Pakistan [11, 12]. In addition, reports from about 30 countries in Africa, Middle East and Asia have identified a relationship between the geographic pattern of distribution of the CCHF disease and the distribution of *Hyalomma* tick vectors [13].

Furthermore, most CCHF cases are the result of occupational exposure among abattoir workers, farmers, shepherds, veterinarians, laboratory workers and healthcare workers. During a nosocomial outbreak at a hospital in South Africa, 33 % of medical personnel exposed via needle stick injuries became ill while approximately 9 % of those who had other forms of contact with infected blood also developed CCHF [14].

Previous CCHF seroprevalence studies among pastoralists, healthy populations in endemic communities as well as in outpatient settings have provided valuable information on the disease epidemiology including risk factors and hot spots which have enabled a more focused preventive approach towards limiting the spread of this disease in many countries [10, 15]. As a result, surveillance for CCHFV in human and vector populations provides an opportunity to monitor the likelihood of a disease of potentially severe impact in humans [16].

As part of a broader study examining occupational risk of exposure to vector-borne and zoonotic pathogens in a high-risk abattoir worker population in Ghana, hard ticks were collected from freshly slaughtered livestock and tested for CCHFV. In addition, human blood samples were collected and tested for CCHF virus specific IgM and IgG antibodies.

## Methods

Samples were collected once a month over a 6-month period at the Kumasi Abattoir in the Ashanti Region of Ghana, spanning May to November 2011.

Freshly slaughtered animals including cattle, goat and sheep were examined for the presence of hard ticks. Ticks were removed from the freshly slaughtered animals, using blunt forceps and placed in cryogenic vials containing RNAlater® solution to maintain intact RNA during shipment.

All samples were transported to US Naval Medical Research Unit No. 3 in Cairo, Egypt, for taxonomic identification using the African Ixodidae key [3]. After identification, the ticks were grouped into pools by species, sex, and animal source. Ticks pools with extraction Lysis buffer and specific beads were homogenized by shaking in Mini-Beadbeater-96 (BioSpec, Bartlesville, OK, USA). RNA was extracted following QIAamp Viral RNA Kit (Qiagen, Valencia, CA) protocol [17]. CCHFV S segment was detected by specific primers and MGB probe established by Garrison et al. [18] Reverse transcription PCR was performed using SuperScript® One-Step RT-PCR System with Platinum® *Taq* DNA Polymerase (Invitrogen, Carlsbad, CA, USA).

Animal handlers who provided consent to participate in the study were asked to provide a baseline blood sample at the first monthly visit. During monthly visits from 2 to 5 months following initial sampling, blood samples were obtained from animal handlers who reported fever during the preceding 30 days. At 6 months following study initiation, all animal handlers who provided baseline blood sample were invited to provide a follow-up blood sample.

The blood samples collected were tested for a panel of vector borne and zoonotic pathogens including CCHF, by serology at the Department of Virology, Noguchi Memorial Institute for Medical Research and confirmed at the virology department of the US Naval Medical Research Unit No. 3, Cairo, Egypt.

The CCHF virus specific IgM and IgG antibodies test was performed using a commercially available kit, which came with its own internal negative and positive controls (VectoCrimean-CHF-IgG and IgM ELISA test kits; Vector-Best, Novosobirsk, Russia). Demographic and epidemiological data were obtained from enrolled participants using a structured questionnaire.

Data was managed using Microsoft Access program and analyzed using the Statistical Package for Social Sciences (SPSS) version 20. All statistical tests were conducted at a 95 % confidence level.

## Results

A total of 428 freshly slaughtered animals were examined: 149 (34.8 %) cattle, 149 (34.8 %) goat and 130 (30.4 %) sheep. In total, 144 ticks were identified from 57 (13.3 %) of the animals: 52 (34.9 %) cattle, 4 (3.1 %) sheep and 1 (0.7 %) goat.

Of the 52 tick laden cattle, 31 (59.6 %) were local breeds while 19 (36.5 %) were imported from Burkina Faso, 1 (1.9 %) from Mali, and 1 (1.9 %) from Cote d’Ivoire. Additionally, 3 (75 %) of the sheep were local breeds while 1 (25 %) was imported from Burkina Faso. The only goat with ticks in our study was imported from Niger.

The ticks identified in this study were all from the family Ixodidae; majority, 93 (64.5 %), of which belonged to the genus *Ambylomma*. This included the single tick obtained from the goat and most (60.8 % and 88.9 %) of those retrieved from cattle and sheep respectively.

In addition, 27 (18.8 %) of the ticks belonged to the genus *Hyalomma*, comprising 20 % of those retrieved from cattle and 10.1 % of those from sheep. The remaining ticks, 24 (16.7 %), belonged to the genus *Boophilus* and were all collected from cattle.

Of 97 tick pools tested (144 ticks of the genera *Ambylomma*, *Hyalomma* and *Boophilus*), 5 pools were positive for CCHFV (Table 1). All 5 pools contained ticks collected from local cattle breeds. The composition of the 5 positive pools was as follows: One pool comprised female *H. excavatum*, 2 comprised female *A. variegatum* while 2 other pools comprised male *A. variegatum;* all proven vectors of CCHF [1, 5, 6]. All positive pools were comprised of adult ticks.

**Table 1:** Genera and species of 5 positive adult tick pools from which CCHFV was isolated

Number of tick pools and sex (M/F) of ticks in pools	Genera and species of Ixodidae ticks in various tick pools	
	Ambylomma	Hyalomma
1 (F)	Nil	H. excavatum
2 (F)	A. variegatum	Nil
2 (M)	A. variegatum	Nil

A total of 109 animal handlers provided consent to participate in the broader study examining occupational risk of exposure to vector-borne and zoonotic pathogens in the high-risk abattoir worker population in the Ashanti region of Ghana, out of which 108 (comprising 6 (5.6 %) females and 102 (94.4 %) males) provided blood sample at baseline.

Twenty (18.5 %) of the animal handlers who provided a baseline blood sample reported acute febrile illness during the follow-up period and provided blood samples. Sixty (55.6 %) of persons who provided baseline sample also provided blood sample at the final visit at 6 months.

From baseline through monthly visit 6, a total of 188 blood samples were collected and tested for CCHF by serology. Seven samples (3.7 %) drawn from six persons were anti-CCHF IgG positive with 1 of the 7 also being anti-CCHF IgM positive. As a result the overall seroprevalence of CCHF identified was 5.7 %.

Of the 7 anti-CCHF IgG positive samples, 4 (including the 1 IgM positive sample) were collected at baseline while three were collected at the 6^th^ month of sample collection. None of the 6 persons who were anti-CCHF IgG or IgM positive reported a history of fever within a 30-day period prior to sample collection.

The serum sample of one of the three persons whose blood samples were anti-CCHF IgG positive at 6 months was also IgG positive at baseline, while serum samples from the other 2 persons tested negative to both anti-CCHF IgM and IgG at baseline.

All seven anti-CCHF IgG/IgM samples were collected from 6 Ghanaian males within the age range of 35 to 48 years (average age = 39 years). All six persons who were anti-CCHF IgG/IgM positive handled animal parts and were engaged in clean up of the abattoir as part of their work.

## Discussion

Crimean Congo hemorrhagic fever is a considerable public health threat which can have significant effect on abattoir workers and healthcare personnel, especially in resource-poor countries [9]. The potential of CCHF to cause nosocomial outbreaks, and the limited options available for treatment and management of infected persons underscore the need for surveillance [10]. The detection of CCHFV in proven vectors of CCHF in our study, supports surveillance in abattoir workers who often may be exposed to infected ticks as well as blood and tissues of infected livestock.

The overall seroprevalence of CCHF IgG antibodies, which is indicative of prior exposure to CCHV in our study was 5.7 %. Serology results obtained after testing 188 blood samples from animal handlers at the abattoir suggest previous and active circulation of CCHF in the slaughterhouse workers.

Four of the seven anti-CCHF IgG samples identified in our study were detected at baseline while the remaining three were detected from samples collected at monthly visit 6. The detection of CCHF IgG positives among 3 (4.4 %) of the samples collected at monthly visit 6 out of which two samples were from people whose serum samples neither tested positive for CCHF IgG nor IgM at baseline, suggest that those two persons may have had exposure to the CCHF virus after the baseline visit.

Although none of the 7 anti-CCHF IgG/IgM positives observed in this study reported acute febrile illness during a 30 day period prior to their sample collection dates, we are unable to conclude that CCHF virus is not currently causing acute febrile illness among the animal handlers; we relied on self reporting of fever among the animal handlers.

The dominant tick collected from Ghanaian cattle is the three-host *A. variegatum,* which is one of the most harmful ticks in Africa [19]. The fact that CCHFV positive pools are present in ticks collected from cattle must be considered a threat to human health, since pathogens can be disseminated through other hosts including wild animals and birds.

The results of this study also suggest that, while meat and meat products serve as an essential source of protein, trade in live animals, meat, and meat products can also serve as a mobile pool of diseases such as CCHF, with potentially large economic and health effects [16].

Furthermore, the influence of climatic conditions on tick activity and occurrence of CCHF have been identified in other studies. Future studies focusing on understanding the relationship between climatic conditions and the occurrence of CCHF could be helpful in the establishment of early warning systems in the surveillance of this disease [12].

**Fig. 1:**
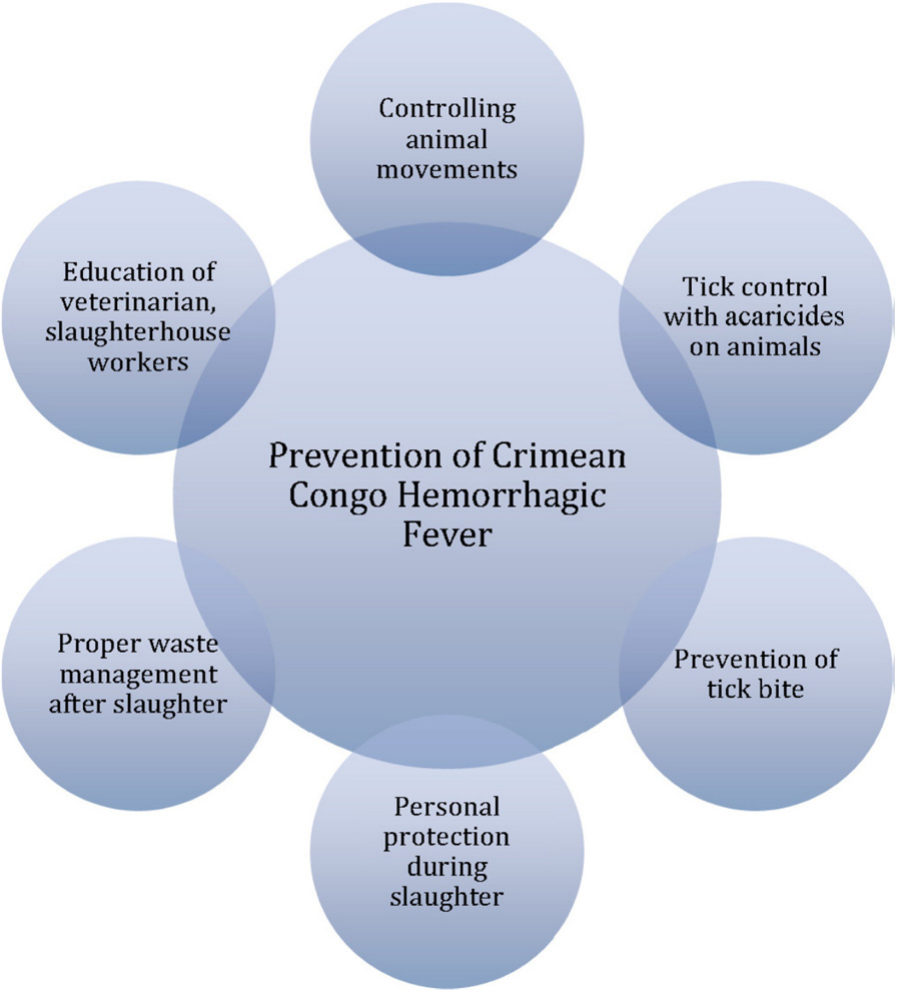
Major components of prevention of Crimean-Congo Hemorrhagic Fever [20]

In addition, as illustrated in Fig. 1, implementation of some CCHF risk mitigating measures such as the avoidance of tick bites, use of personal protective equipment and control of CCHF in animals by using acaricides in livestock production facilities could avert a CCHF outbreak [20].

## Conclusions

This study detected human exposure to CCHF virus in a high risk abattoir population and also identified the CCHF virus in proven tick vectors in Ghana. The CCHFV was detected in ticks collected from cattle, one of the livestock known to play a role in the amplification of the CCHF virus.

While future studies targeted at understanding the relationship between climatic conditions and the occurrence of CCHF could be helpful in the establishment of early warning systems in the surveillance of this disease, implementation of CCHF risk mitigation measures could avert an outbreak of the CCHF disease in Ghana.

## Abbreviations

CCHF, crimean-congo hemorrhagic fever; CCHFV, crimean-congo hemorrhagic fever virus; ELISA, enzyme linked immunosorbent assay
